# Supplementary material for: Evolution and subfunctionalization of CIPK6 homologous genes in regulating cotton drought resistance
Source: Nat Commun. 2024 Jul 9;15:5733. doi: 10.1038/s41467-024-50097-3 (PMC11231324; doi:10.1038/s41467-024-50097-3)
Supplement: Supplementary file 1 — Supplementary Information [file 41467_2024_50097_MOESM1_ESM.pdf]

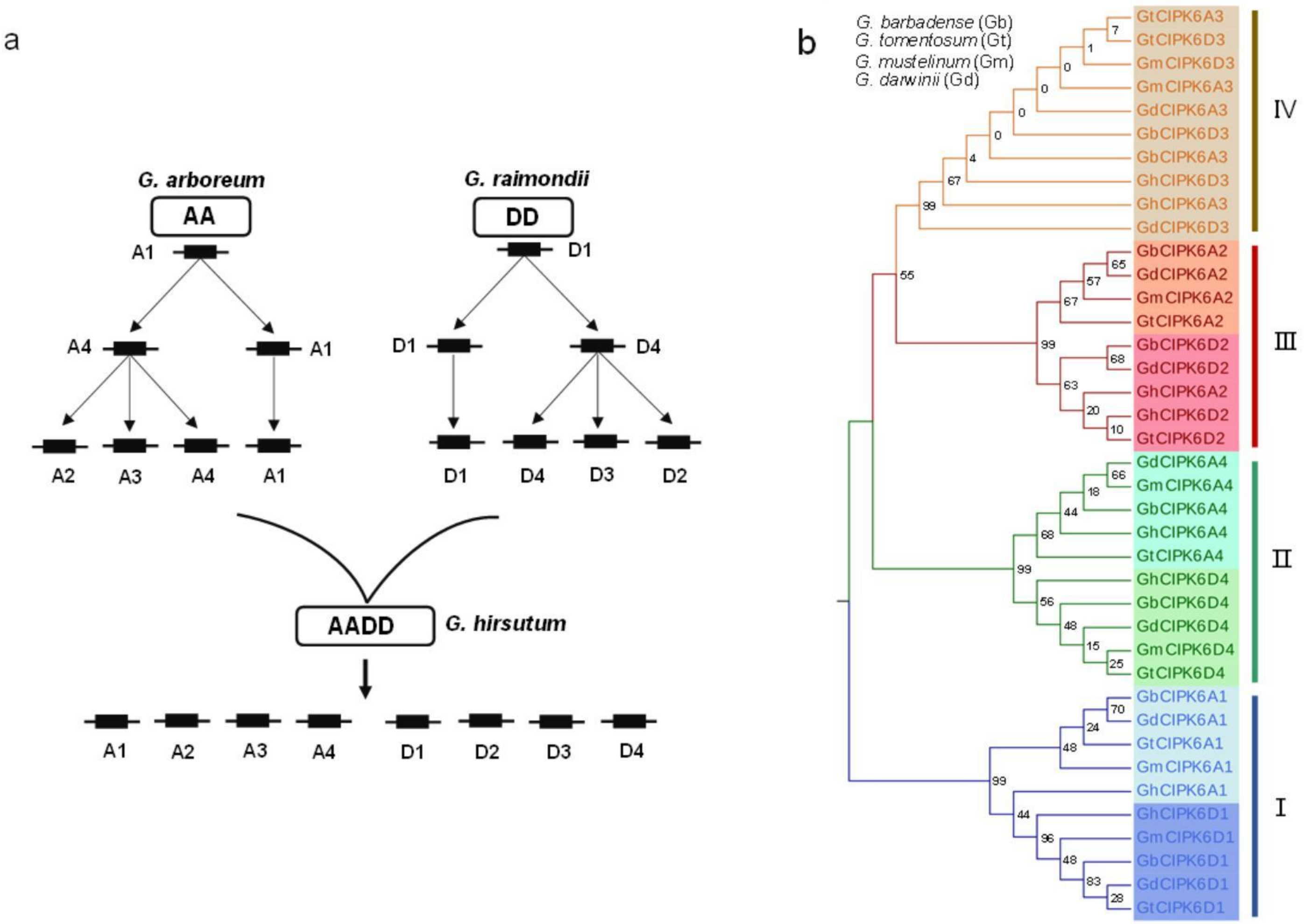

Supplementary Fig. 1. Simulation of the evolution of *CIPK6* gene family and analysis of evolution in other tetraploid cotton genus.

a, Evolutionary order model of eight *GhCIPK6* genes after tetraploidy. b, Phylogenetic tree of eight *CIPK6* genes in different tetraploid *Gossypium*. The different numbers at the node of the evolutionary tree are the bootstrap values. They are used for evaluating the reliability of branches in evolutionary trees.

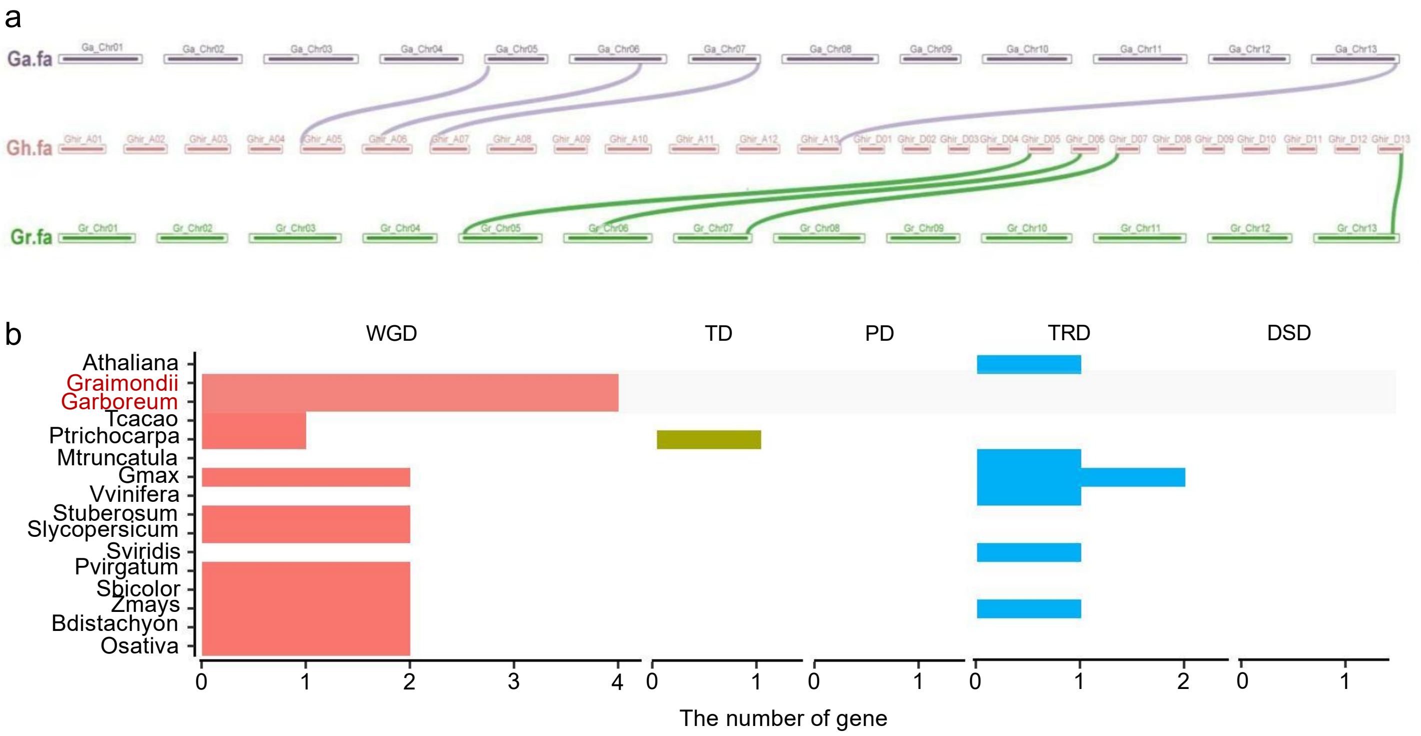

Supplementary Fig. 2. Analysis of gene synteny and duplication of *CIPK6s*.

a, Chromosomal synteny analysis of eight *CIPK6* genes among *G. raimondii* (purple), *G. aboreum* (brown) and *G. hirsutum* (green). **b**, The number of CIPK6 genes derived from different modes of duplication in 16 plant genomes (including *G. aboreum* and *G. raimondii*). WGD whole-genome duplication, TD tandem duplication, PD proximal duplication, TRD transposed, DSD dispersed duplication.

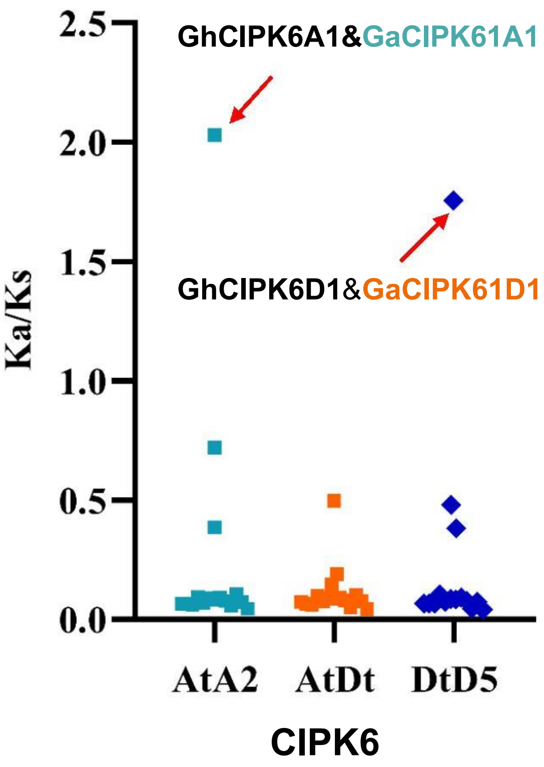

Supplementary Fig. 3. Ka/Ks analysis of *CIPK6* genes in *G. raimondii* (D5), *G. aboreum* (A2) and *G. hirsutum* (At/Dt).

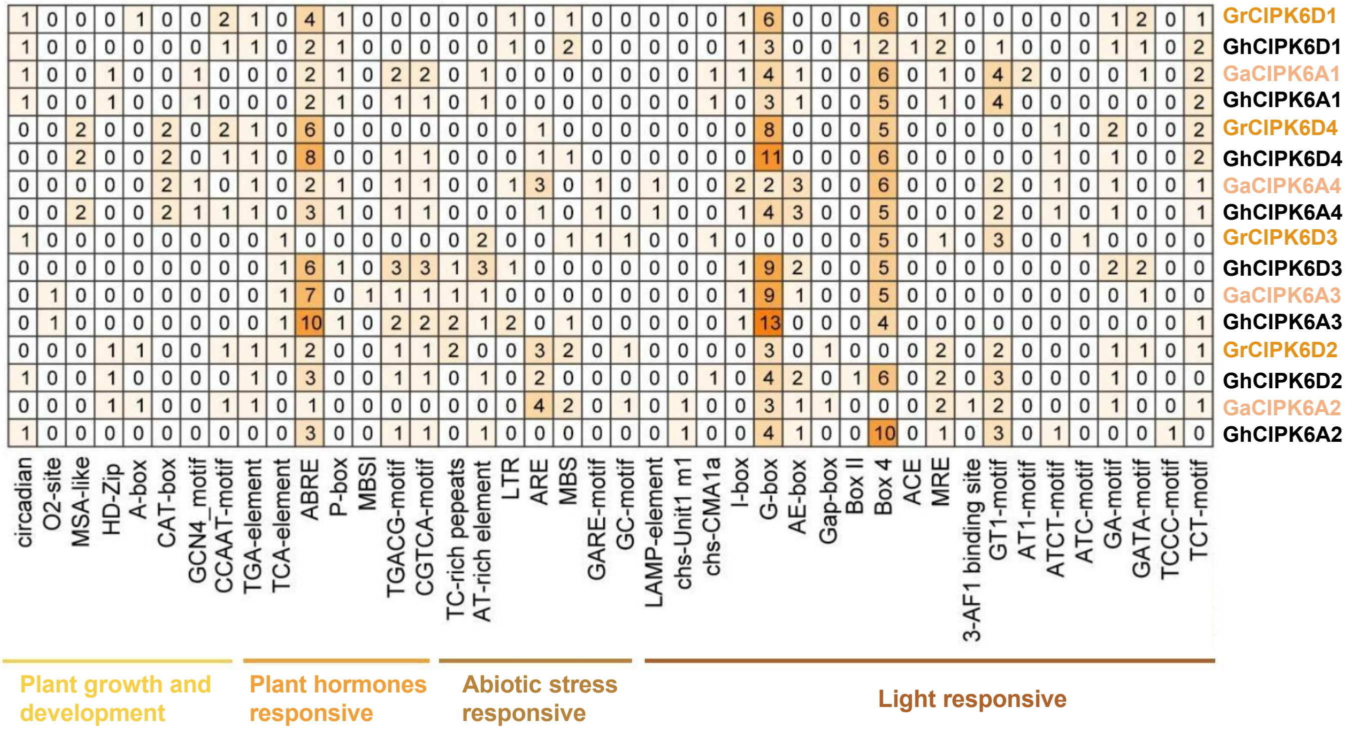

Supplementary Fig. 4. Analysis of cis-acting elements on the promoter of *CIPK6* in *Gossypium*. Brown for light response element, gray for abiotic stress response element, orange for plant hormones response element, yellow for plant growth and development related element.

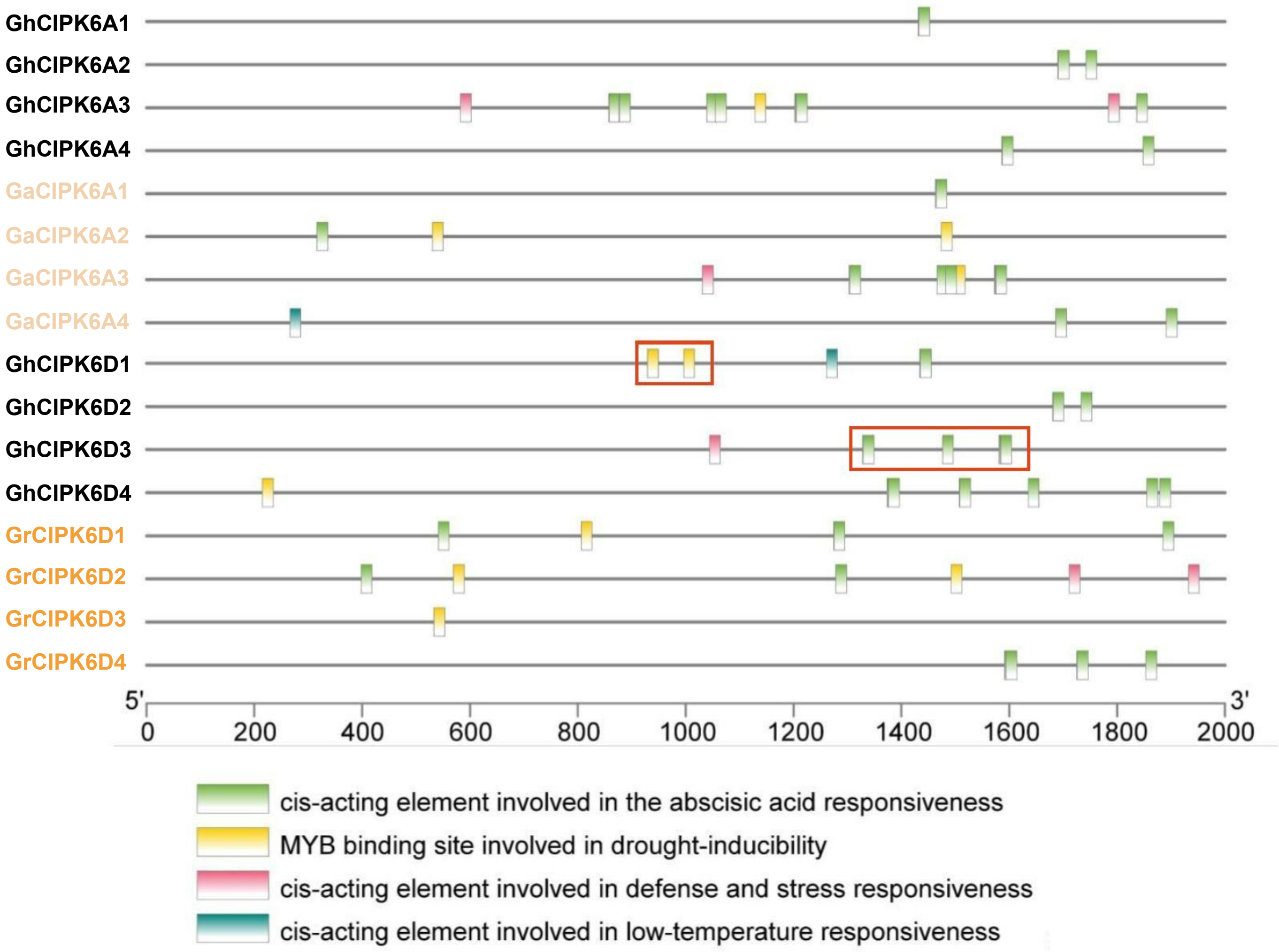

Supplementary Fig. 5. Analysis of cis-active elements related to drought response on the promoter of *CIPK6* genes among *Gossypium*.

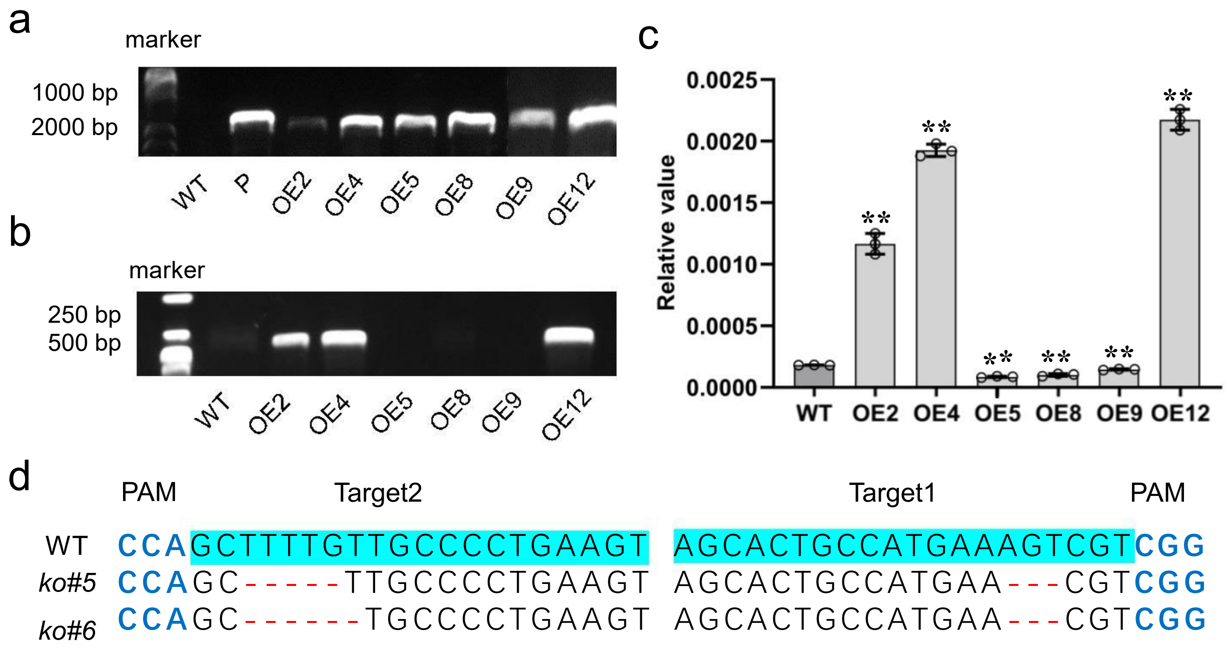

Supplementary Fig. 6. Molecular identification of *GhCIPK6D1* transgenic plants.

a, Positive identification of overexpressing *GhCIPK6D1* transgenic lines. **b**, Expression analysis of *GhCIPK6D1* in transgenic lines by RT-PCR. **c**, Expression analysis of *GhCIPK6D1* in transgenic lines by RT-qPCR. Data are means±SD (*n*= 3 biological replicates). Significant difference analysis used two-tailed Student's t test (***P*<0.01). **d**, Sequence analysis of *GhCIPK6D1* mutants *ko#5* and *ko#6* at the target sites. The PAM motif is marked in blue. The red dotted line represents deletion compared with WT. Source data are provided as a Source Data file.

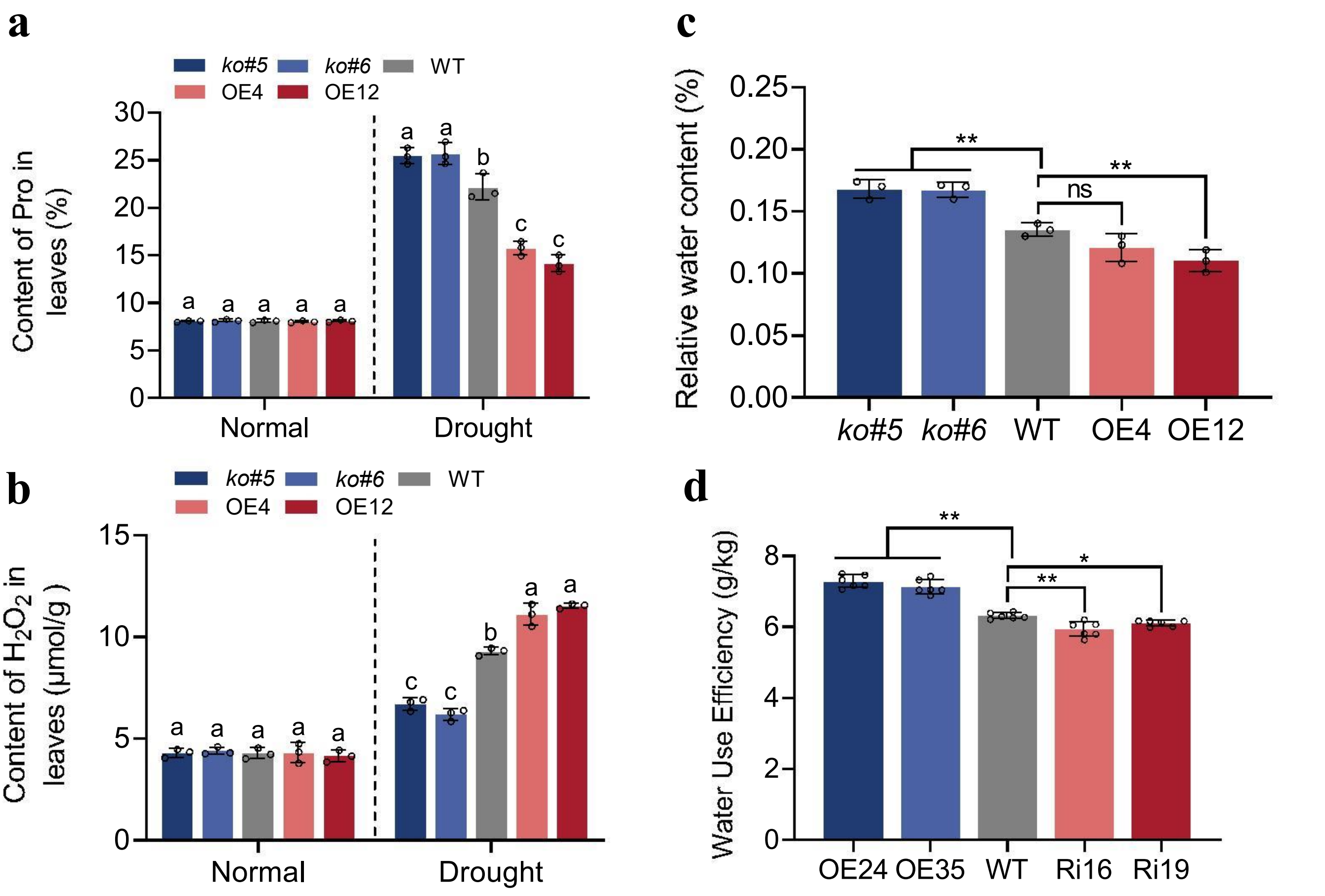

Supplementary Fig. 7. Determination of drought-related indicators between *GhCIPK6D1* and *GhCIPK6D3* transgenic and WT plants.

a-b, The content analysis of proline (a) and H_2O_2 (b) between *GhCIPK6D1* transgenic lines and WT plants. Data are means ± SD (*n* = 3 biological replicates). Different letters above the columns of each compartment indicate a significant difference at $P < 0.05$ (one-way ANOVA followed by Duncan's multiple range test). **c**, Relative water content analysis between *GhCIPK6D1* transgenic lines and WT plants. Data are means ± SD (*n*= 3 biological replicates). Significant difference analysis used one-way ANOVA followed by Duncan's multiple-range test (***P*<0.01), and ns indicates no significant difference. **d**, The Water Use Efficiency analysis between *GhCIPK6D3* transgenic lines and WT plants. Data are means±SD (*n*= 6 biological replicates). Significant difference analysis used one-way ANOVA followed by Duncan's multiple-range test (***P*<0.05, ***P*<0.01). Source data are provided as a Source Data file.

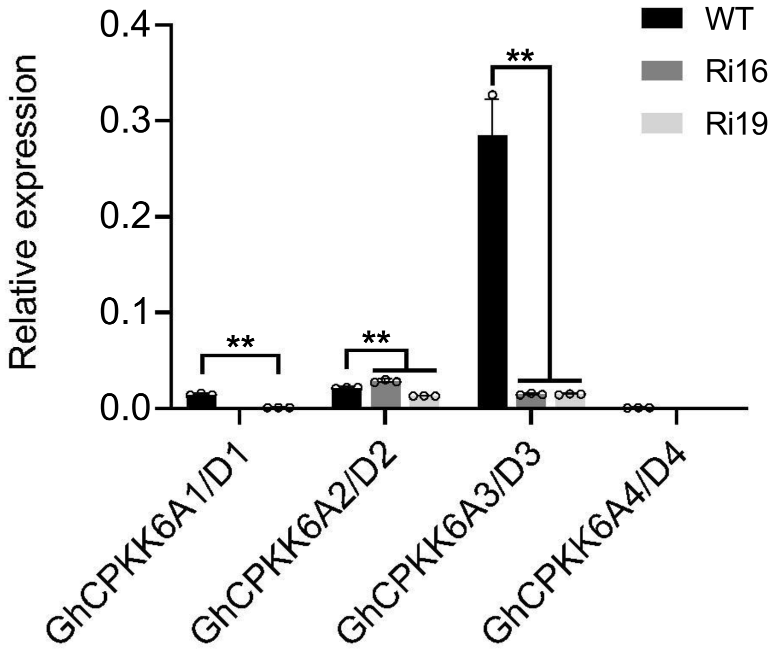

Supplementary Fig. 8. Analysis of expression of *GhCIPK6* homologous genes in Ri16 and Ri19 plants.

Data are means±SD (*n*= 3 biological replicates). Significant difference analysis used one-way ANOVA followed by Duncan's multiple-range test (***P*<0.01). Source data are provided as a Source Data file.

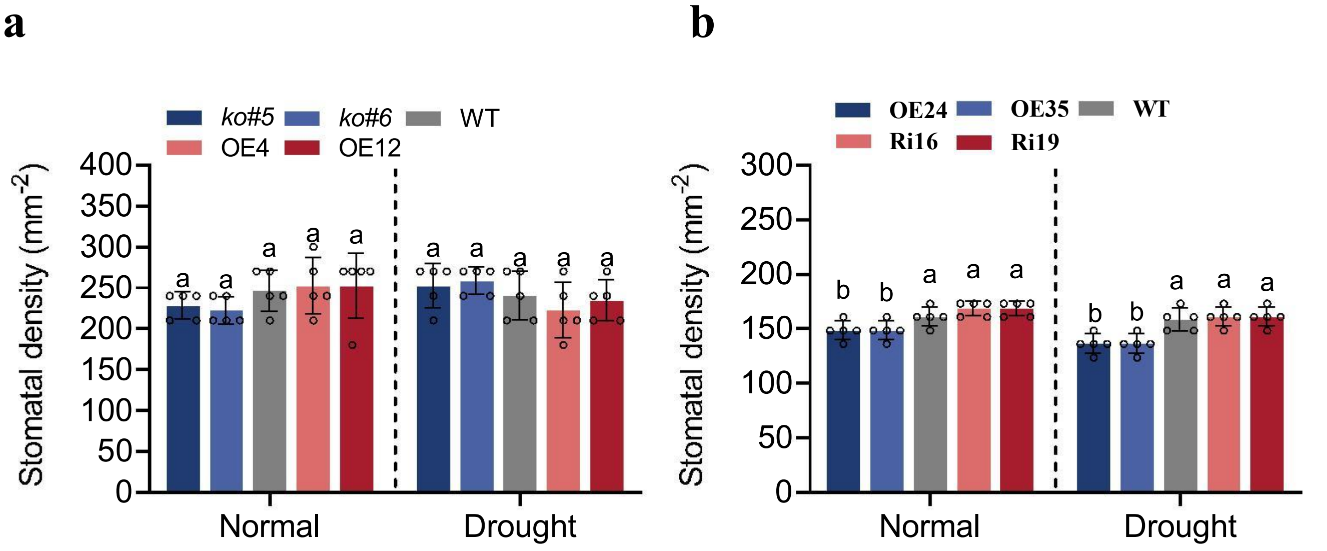

Supplementary Fig. 9. Analysis of stomatal density in transgenic and WT lines under normal and drought stress.

a, Analysis of stomatal density in GhCIPK6D1 transgenic lines. **b,** Analysis of stomatal density in GhCIPK6D1 transgenic lines. Data are means±SD (*n*= 5 biological replicates). Different letters above the columns of each compartment indicate a significant difference at $P < 0.05$ (one-way ANOVA followed by Duncan's multiple range test). Source data are provided as a Source Data file.

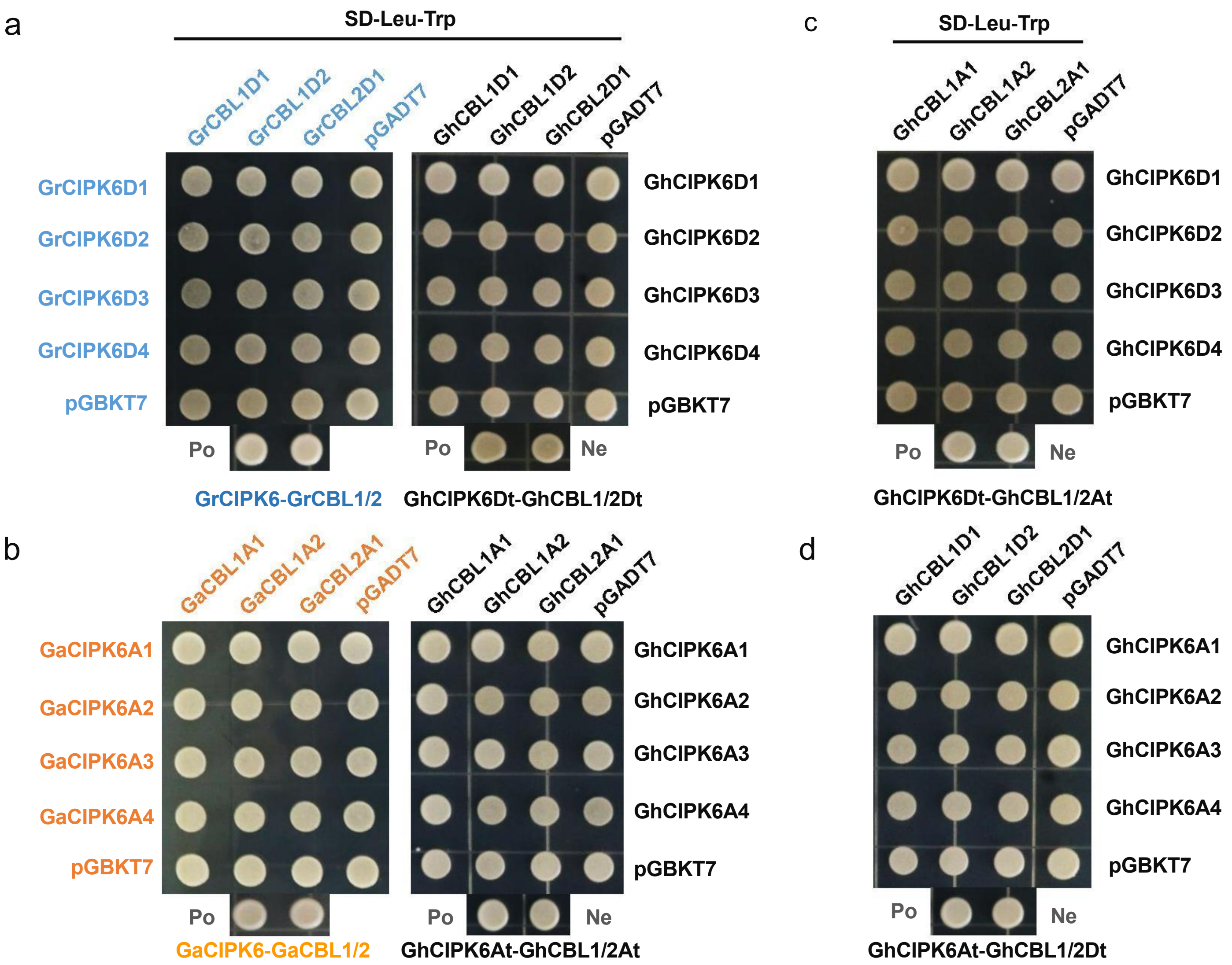

Supplementary Fig. 10. Interaction pattern analysis between CIPK6s and CBL1/2s in *Gossypium* on SD-Leu-Trp medium.

a, Interaction between *G. raimondii* and upland cotton Dt subgenome. **b**, Interaction between *G. arboretum* and upland cotton At subgenome. **c**, Interaction between At subgenome of GhCBL1/2s and Dt subgenome of GhCIPK6s in upland cotton. **d**, Interaction between Dt subgenome of GhCBL1/2s and At subgenome of GhCIPK6s in upland cotton. The blue front indicates, the orange front indicates *G. arboretum*. "Po" represents positive control and "Ne" represents negative control. The positive control was the diploid hybrid yeast containing pGBKT7-53 and pGADT7-T, the negative control was the diploid hybrid yeast containing pGBKT7-Lam and pGADT7-T.

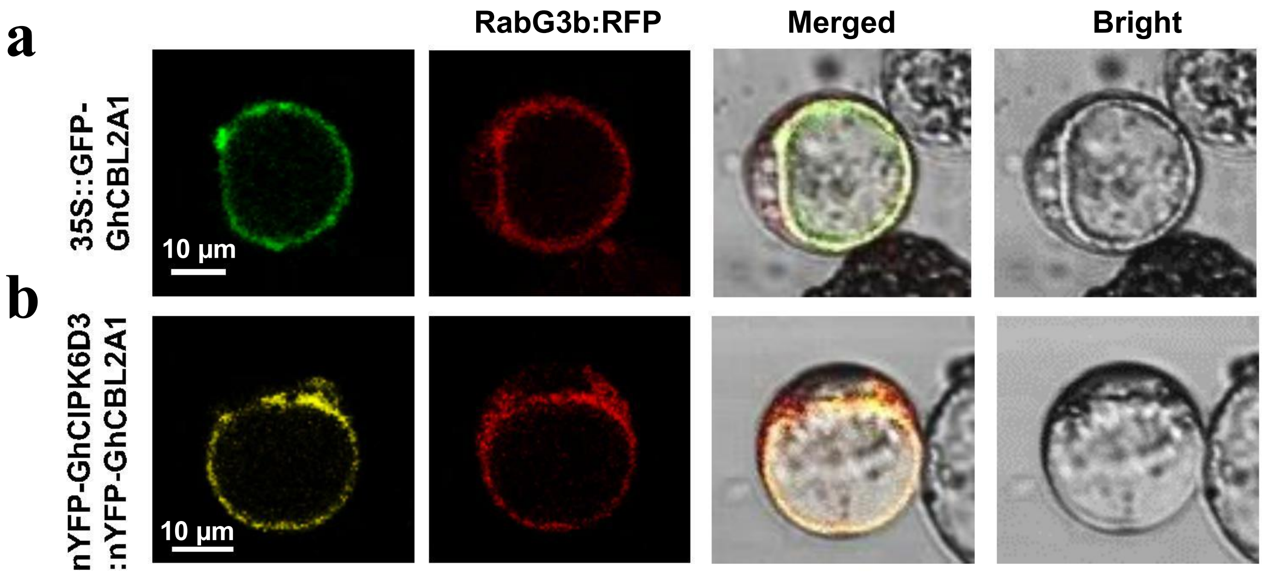

Supplementary Fig. 11. Subcellular localization of GhCBL2A1 and interaction with GhCIPK6D3 in cotton protoplasts.

a, Subcellular localization of GhCBL2A1 in cotton protoplasts. **b**, Interaction between GhCBL2A1 and GhCIPK6D3 by BiFC assays in cotton protoplasts. RabG3b (a protein that has been reported to be localized in the vacuole membrane) was used as the vacuole membrane marker. Bars = 10 μm.

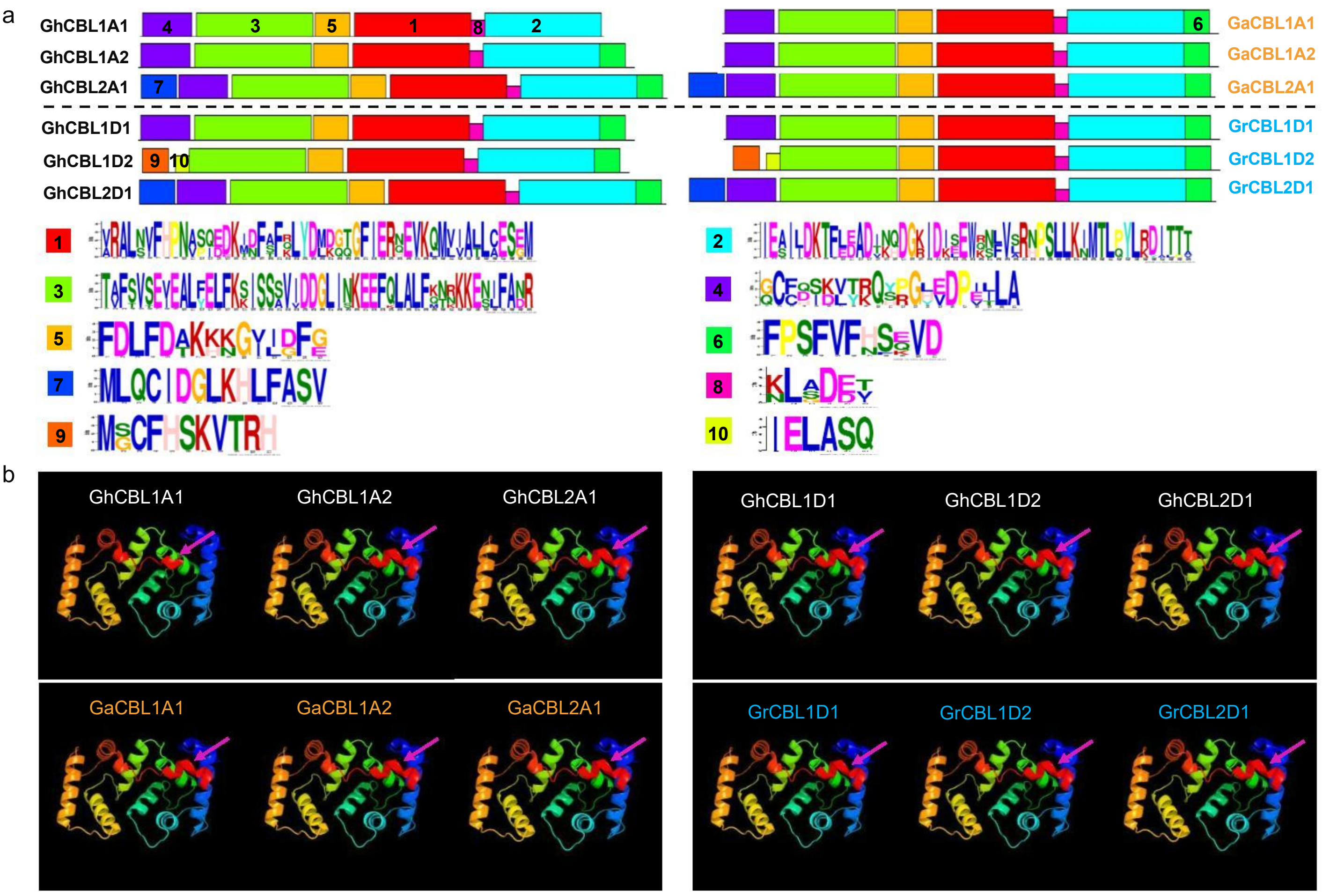

Supplementary Fig. 12. Comparative analysis of conserved motif and prediction of 3D protein structure for each pair of CBL1/2s in *Gossypium*.

a, Analysis of conserved domain and motif of amino acid of CBL1/2s. **b**, Analysis of 3D protein structure prediction of CBL1/2s.

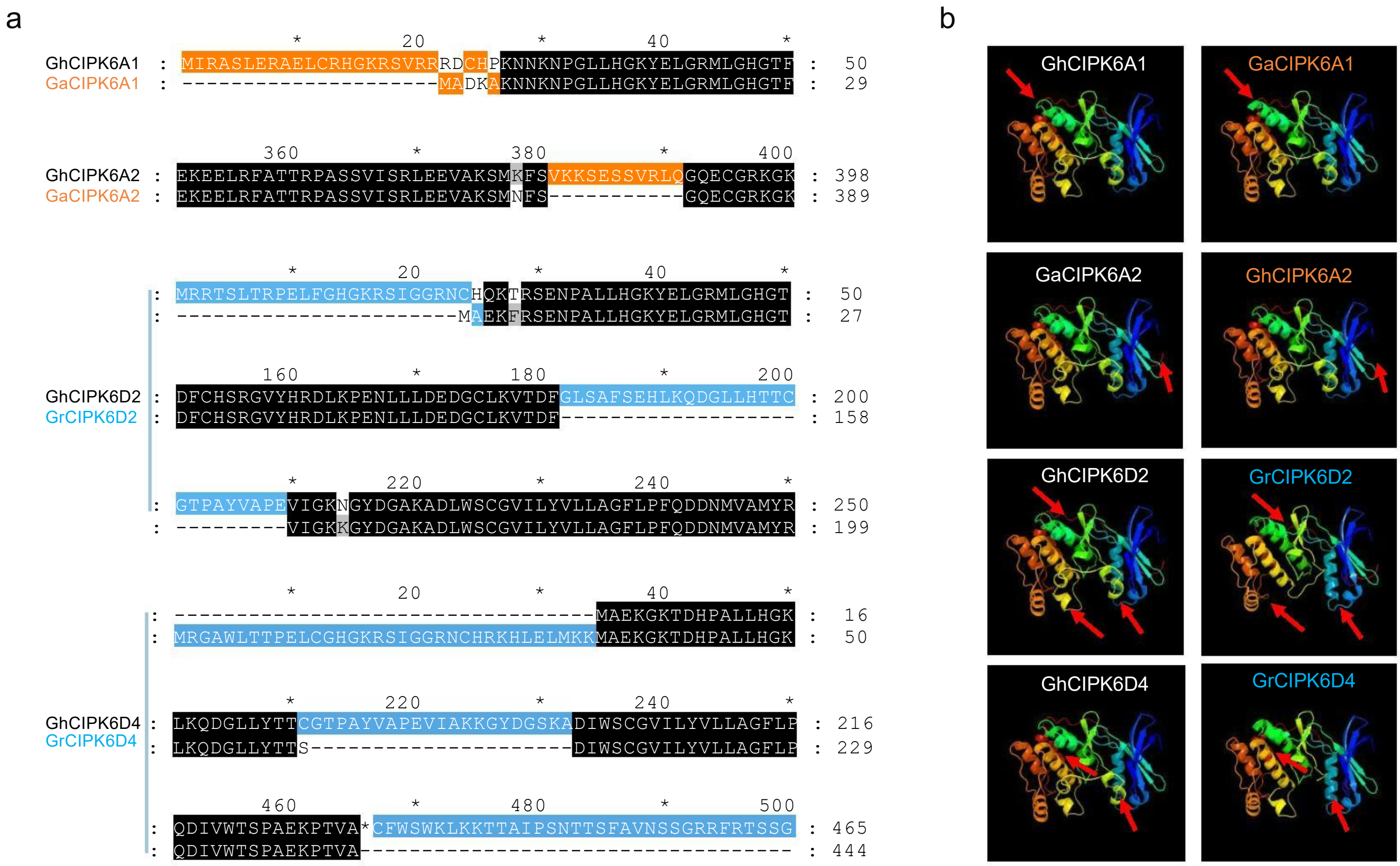

Supplementary Fig. 13. Comparative analysis of amino acid sequences and 3D protein structure of four pairs of CIPK6 in *Gossypium*.

a, Each pair of CIPK6 amino acid sequences were compared and analyzed. Orange and blue background sequences indicate differences. **b**, Analysis of 3D protein structure of eight CIPK6 proteins. Red arrows indicate differences.

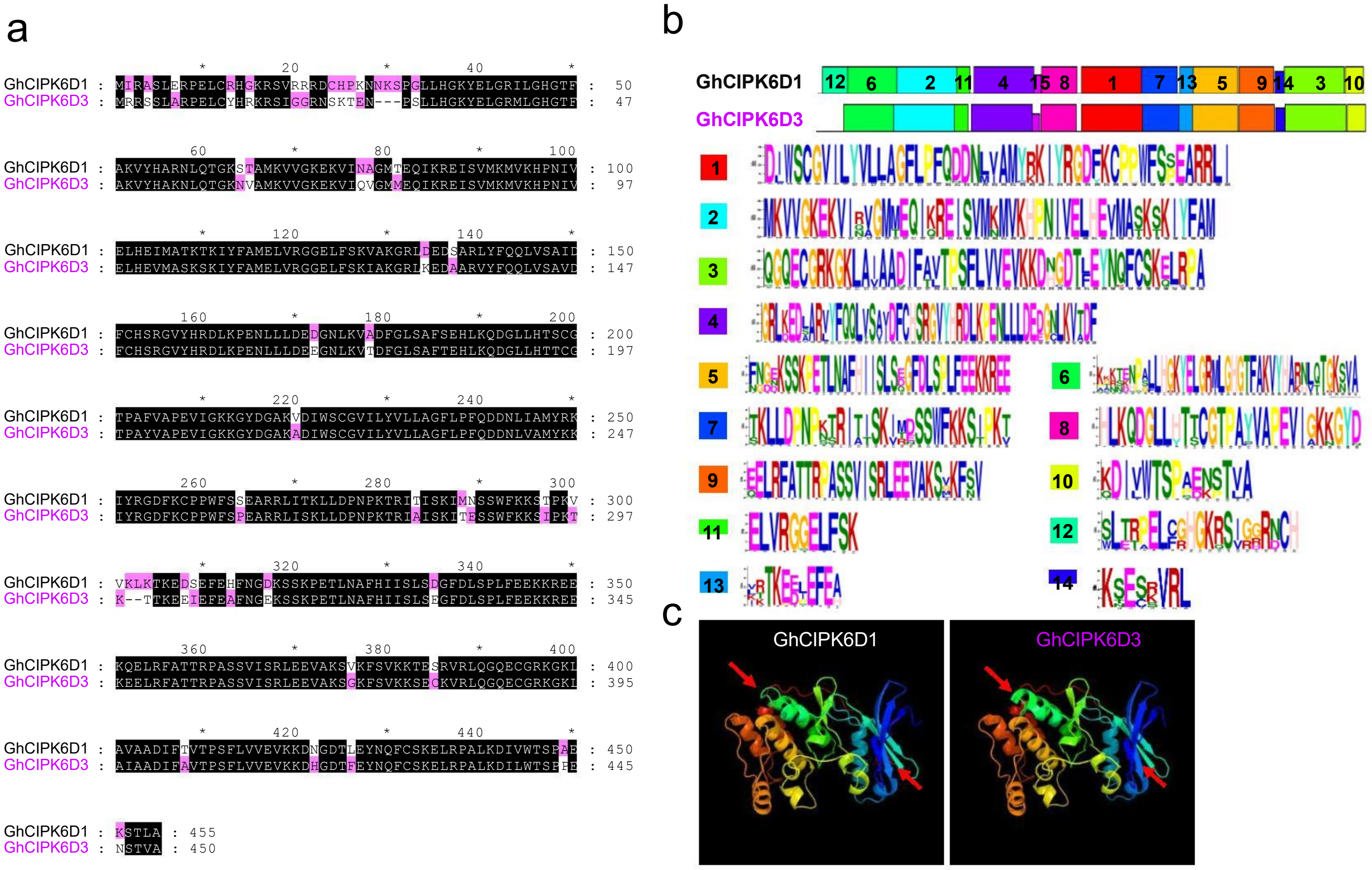

Supplementary Fig. 14. Comparative analysis of amino acid sequences and 3D protein structure between GhCIPK6D1 and GhCIPK6D3.

a, Comparative analysis of amino acid sequence between GhCIPK6D1 and GhCIPK6D3. **b,** Comparative analysis of conserved motif between GhCIPK6D1 and GhCIPK6D3. **c**, Comparative analysis of 3D protein structure between GhCIPK6D1 and GhCIPK6D3. Red arrows indicate differences.

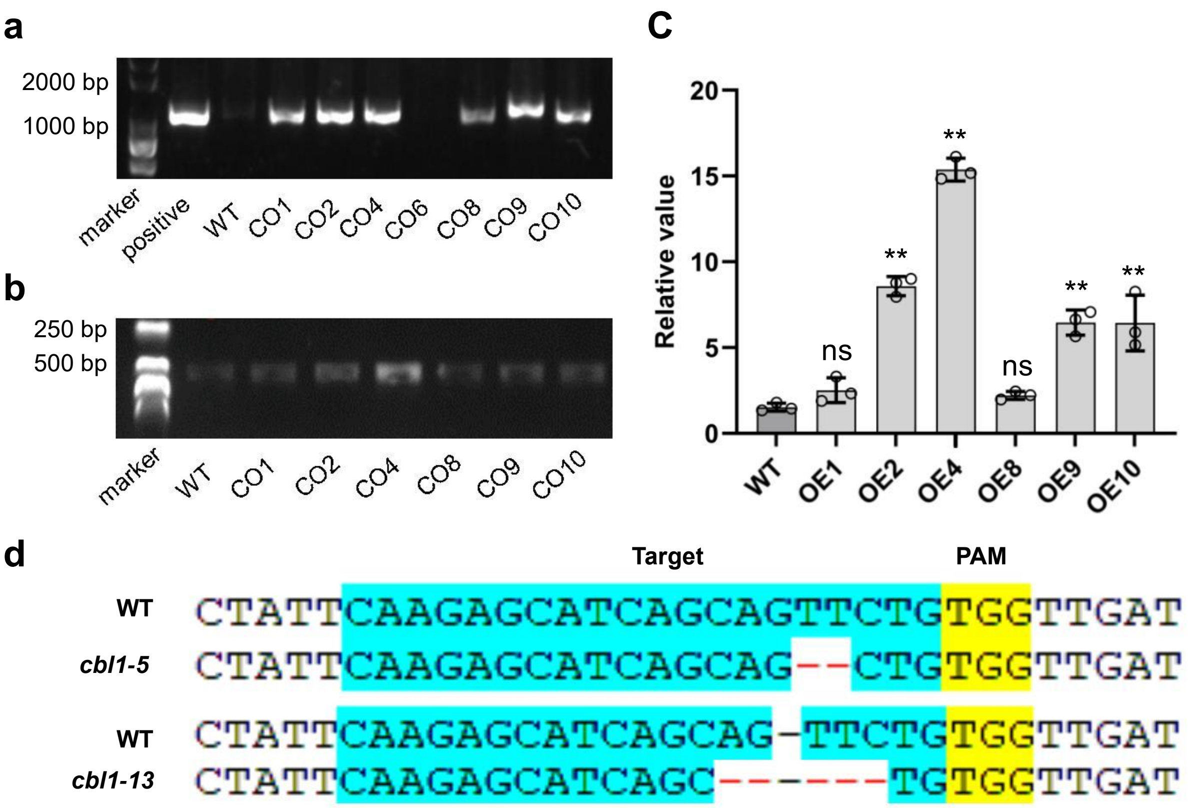

Supplementary Fig. 15. Molecular identification of *GhCBL1A1* transgenic plants.
a, Positive identification of overexpressing *GhCBL1A1* transgenic lines. **b**, Expression analysis of OE-*GhCBL1A1* in transgenic lines by RT-PCR. **c**, Expression analysis of OE-*GhCBL1A1* in transgenic lines by RT-qPCR. Data are means±SD (*n*= 3 biological replicates). Significant difference analysis used two- tailed Student's t test (***P*<0.01), and ns indicates no significant difference. **d**, Sequence analysis of *GhCBL1A1* mutants *cbl1-5* and *cbl1-13* at the target sites. The PAM motif is marked in yellow background. The red dotted line represents deletion compared with WT. Source data are provided as a Source Data file.

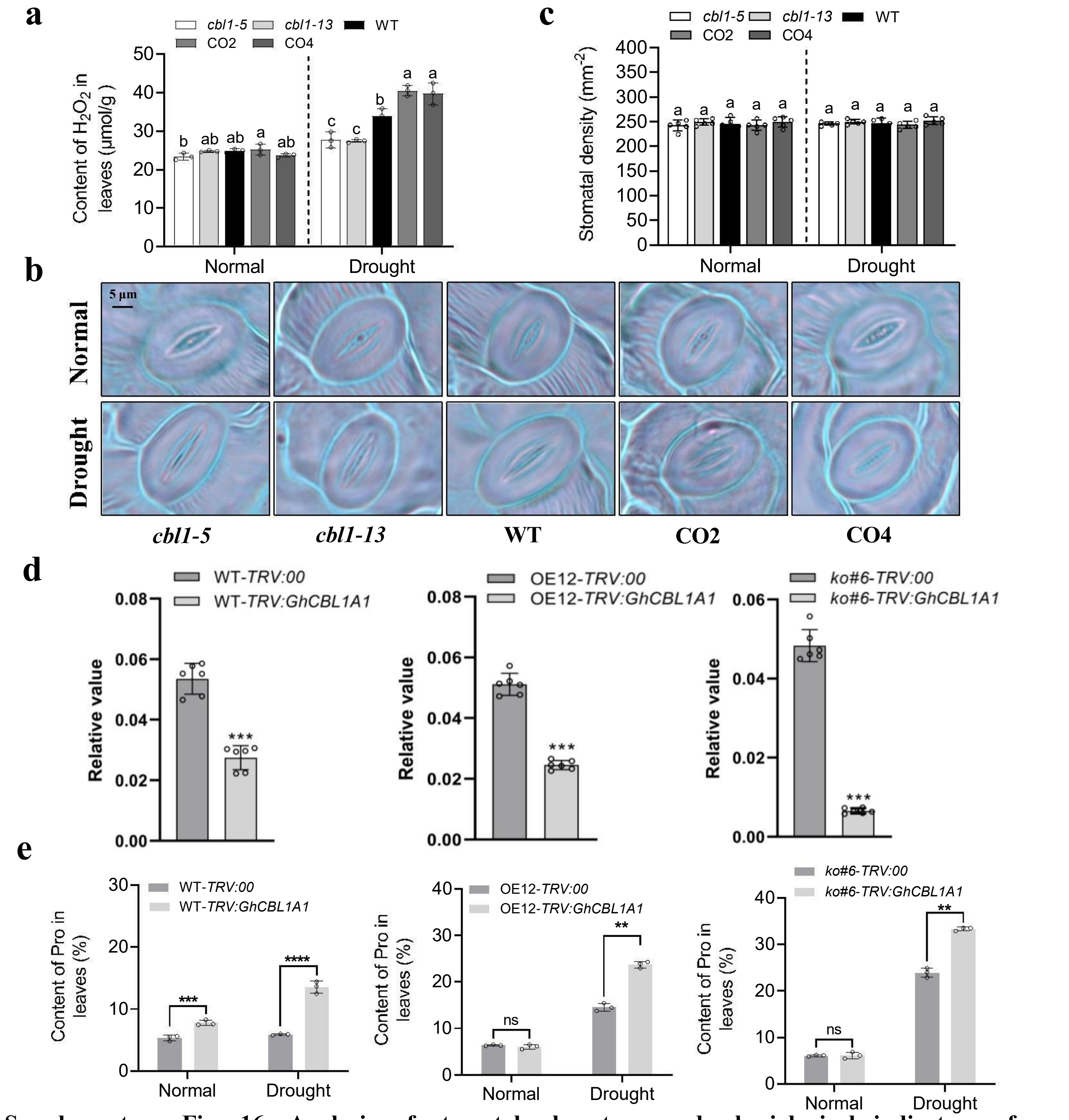

Supplementary Fig. 16. Analysis of stomatal phenotype and physiological indicators of GhCBL1A1-GhCIPK6D1 network in response to drought in cotton.

a, H_2O_2 content in the leaves of transgenic and WT lines under normal and drought stress. Data are means ± SD ($n = 3$ biological replicates). Different letters above the columns of each compartment indicate a significant difference at $P < 0.05$ (one-way ANOVA followed by Duncan's multiple range test). **b,** Light microscopy images of leaf epidermal stomata of transgenic of *GhCBL1A1* and WT lines under normal and drought stress. Bars = 5 μm. **c,** Analysis of stomata density of transgenic of *GhCBL1A1* and WT lines under normal and drought stress. Data are means ± SD ($n = 5$ biological replicates). Different letters above the columns of each compartment indicate a significant difference at $P < 0.05$ (one-way ANOVA followed by Duncan's multiple range test). **d**, Expression analysis of *GhCBL1A1* by VIGS in background of three materials. Data are means ± SD ($n = 3$ biological replicates). Significant difference analysis used two-tailed Student's t test (***P<0.001). **e,** Pro content in the leaves of *GhCBL1A1*-silenced plants in WT, OE12 and *ko#6* backgrounds under normal and drought stress. Data are means ± SD ($n = 3$ biological replicates). Significant difference analysis used two-tailed Student's t test (**P<0.01, ***P<0.001, ***P<0.001), and ns indicates no significant difference. Source data are provided as a Source Data file.

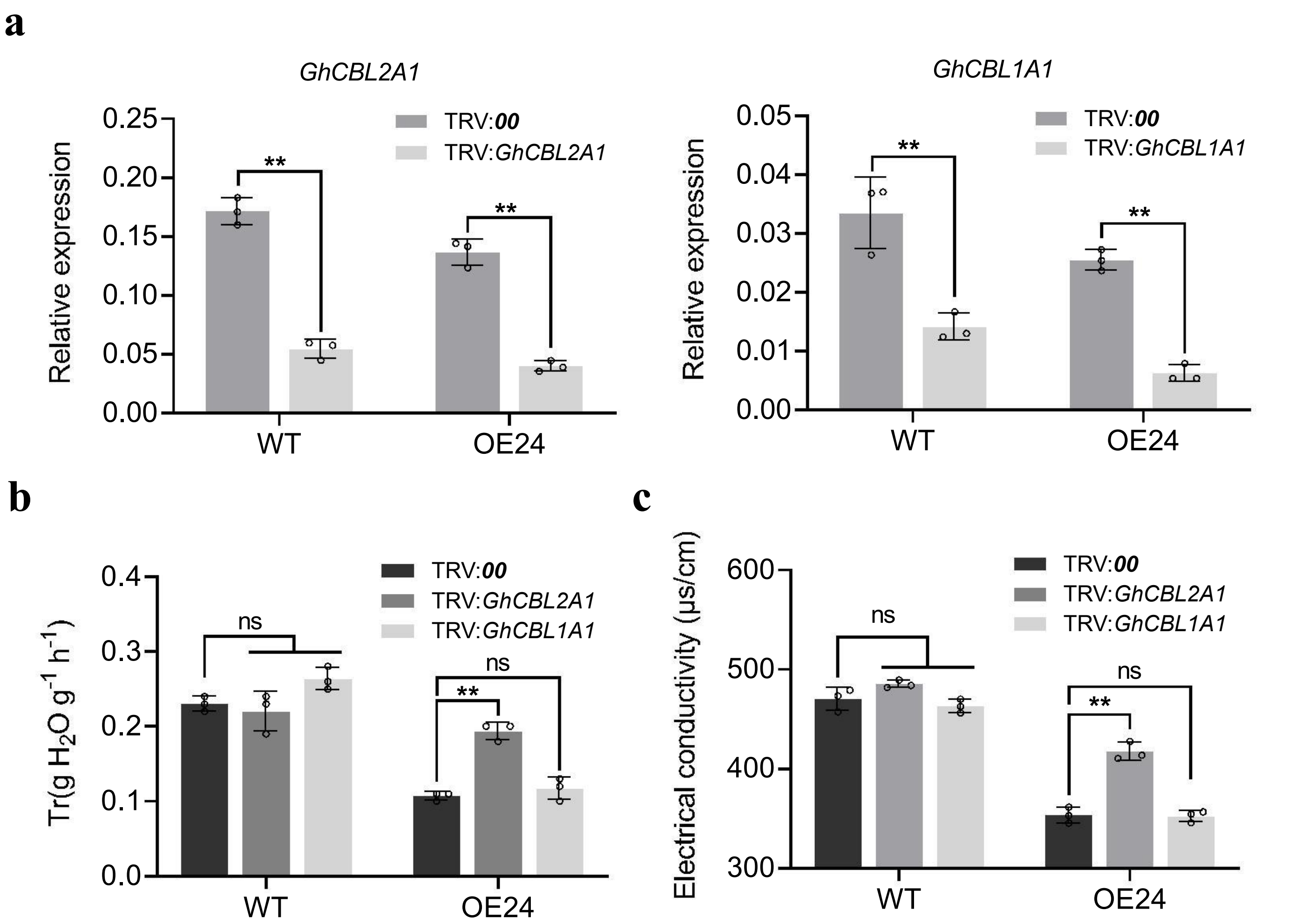

Supplementary Fig. 17. GhCIPK6D3 role in drought response depends on GhCBL2A1 not GhCBL1A1.

a, Relative expression level of *GhCBL1A1* or *GhCBL2A1* in T RV: *GhCBL1A1* or T RV: *GhCBL2A1* and T RV:*00* plants. Background was WT and OE24. Data are means±SD (*n*= 3 biological replicates). Significant difference analysis used two-tailed Student's t test (***P*<0.01). **b**, The transpiration rate of *GhCBL1A1*-silenced or *GhCBL2A1*-silenced plants in the background of WT and OE24 under drought conditions. Plants at four-leaf stage in soil were exposed to drought stress for 10 days. **c**, Relative electrical conductivity of VIGS cotton leaves under drought stress condition. Data in (b) and (c) are means ± SD (*n* = 3 biological replicates). Significant difference analysis used one-way ANOVA followed by Duncan's multiple range test (***P*<0.01), and ns indicates no significant difference. Source data are provided as a Source Data file.

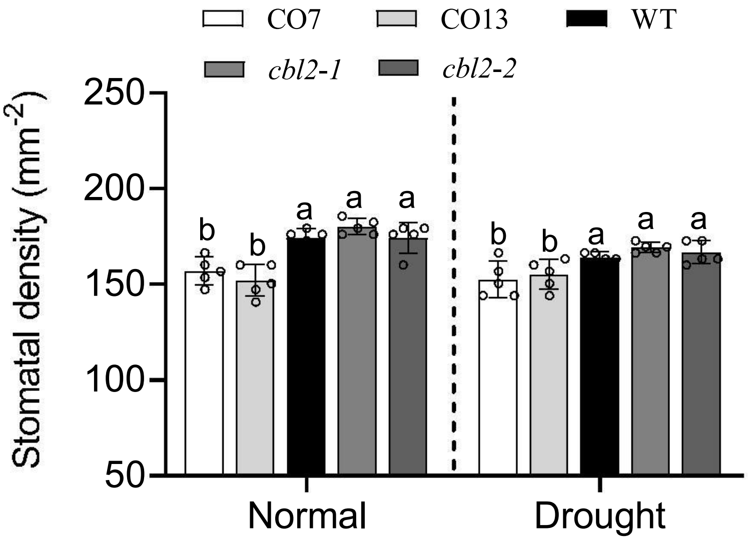

Supplementary Fig. 18. Analysis of stomatal density in transgenic lines and WT plants of *GhCBL2A1* under normal and drought stress.

Data are means±SD (n= 5 biological replicates).Different letters above the columns of each compartment indicate a significant difference at P< 0.05 (one-way ANOVA followed by Duncan's multiple range test). Source data are provided as a Source Data file.

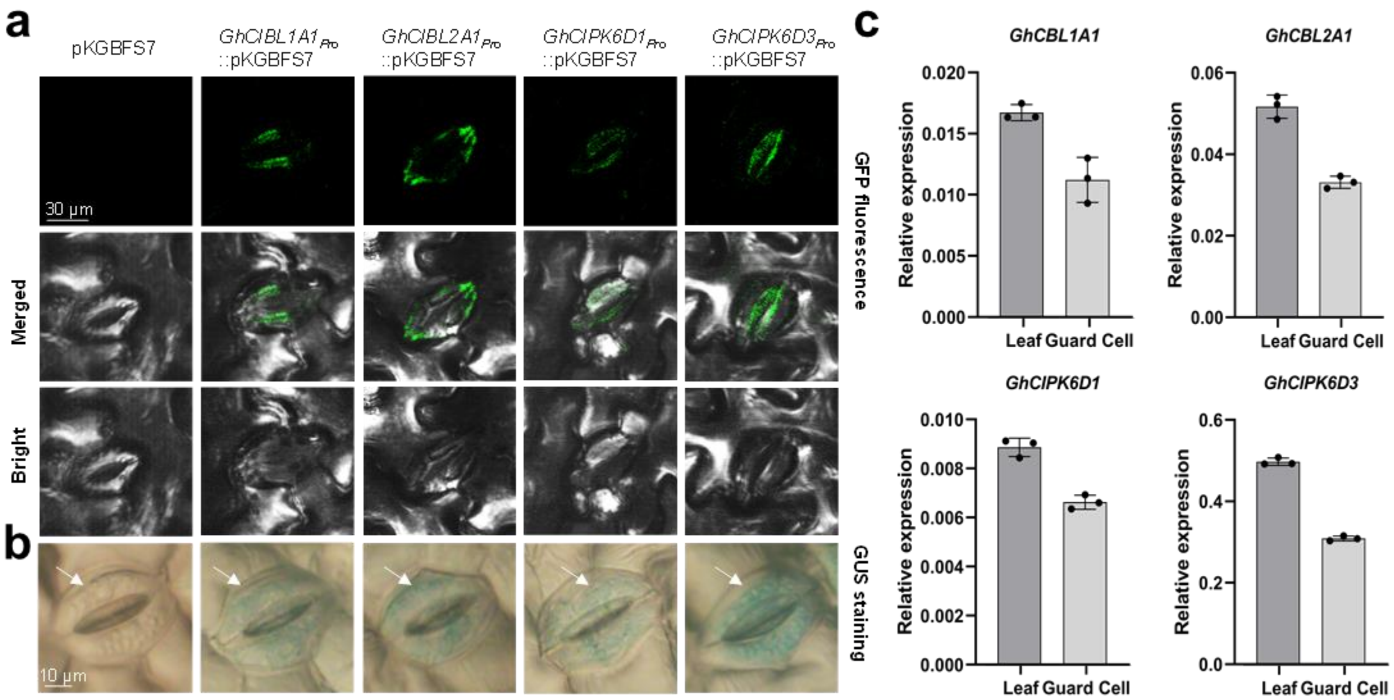

Supplementary Fig. 19. Analysis of expression of *GhCBL1A1*, *GhCBL2A1*, *GhCIPK6D1* and *GhCIPK6D3* in guard cell.

a-b, GFP and GUS protein expression fused with the specific promoter of *GhCBL1A1*, *GhCBL2A1*, *GhCIPK6D1* and *GhCIPK6D3* (**a**). Bars = 30 μm. And GUS staining assays (**b**). White arrows indicate GUS expression in guard cells. Bars = 10 μm. The experiment was independently repeated three times (a-b). **c**, Analysis of expression of *GhCBL1A1/2A1* and *GhCIPK6D1/6D3* in guard cells by qRT-PCR. Data are means ± SD ($n = 3$ biological replicates). Significant difference analysis used two-tailed Student's t test (**P<0.01). Source data are provided as a Source Data file.
