# Supplementary material for: Evolution and subfunctionalization of CIPK6 homologous genes in regulating cotton drought resistance
Source: Nat Commun. 2024 Jul 9;15:5733. doi: 10.1038/s41467-024-50097-3 (PMC11231324; doi:10.1038/s41467-024-50097-3)
Supplement: Supplementary file 3 — Description of Additional Supplementary Files [file 41467_2024_50097_MOESM3_ESM.pdf]

Description of Additional Supplementary Files

File Name: Supplementary Data 1
Description: Information of 23 plant genomes used in this study.

File Name: Supplementary Data 2
Description: Gene, CDS, and promoter sequences of the CIPK6 genes used in this study.

File Name: Supplementary Data 3
Description: Analysis of cis-acting elements of CIPK6 genes promoter in Gossypium

File Name: Supplementary Data 4
Description: List of primers used in this study
