# Supplementary material for: Evolution and subfunctionalization of CIPK6 homologous genes in regulating cotton drought resistance
Source: Nat Commun. 2024 Jul 9;15:5733. doi: 10.1038/s41467-024-50097-3 (PMC11231324; doi:10.1038/s41467-024-50097-3)
Supplement: Supplementary file 8 — Reporting Summary [file 41467_2024_50097_MOESM8_ESM.pdf]

nature portfolio

Corresponding author(s): Xiyan Yang

Last updated by author(s): Jun 13, 2024

Reporting Summary

Nature Portfolio wishes to improve the reproducibility of the work that we publish. This form provides structure for consistency and transparency in reporting. For further information on Nature Portfolio policies, see our Editorial Policies and the Editorial Policy Checklist.

Statistics

For all statistical analyses, confirm that the following items are present in the figure legend, table legend, main text, or Methods section.

n/a	Confirmed	
☐	☒	The exact sample size (*n*) for each experimental group/condition, given as a discrete number and unit of measurement
☒	☐	A statement on whether measurements were taken from distinct samples or whether the same sample was measured repeatedly
☐	☒	The statistical test(s) used AND whether they are one- or two-sided<br>*Only common tests should be described solely by name; describe more complex techniques in the Methods section.*
☒	☐	A description of all covariates tested
☐	☒	A description of any assumptions or corrections, such as tests of normality and adjustment for multiple comparisons
☐	☒	A full description of the statistical parameters including central tendency (e.g. means) or other basic estimates (e.g. regression coefficient) AND variation (e.g. standard deviation) or associated estimates of uncertainty (e.g. confidence intervals)
☐	☒	For null hypothesis testing, the test statistic (e.g. *F*, *t*, *r*) with confidence intervals, effect sizes, degrees of freedom and *P* value noted<br>*Give P values as exact values whenever suitable.*
☒	☐	For Bayesian analysis, information on the choice of priors and Markov chain Monte Carlo settings
☒	☐	For hierarchical and complex designs, identification of the appropriate level for tests and full reporting of outcomes
☒	☐	Estimates of effect sizes (e.g. Cohen's *d*, Pearson's *r*), indicating how they were calculated

Our web collection on statistics for biologists contains articles on many of the points above.

Software and code

Policy information about availability of computer code

Data collection	The 23 sequenced plant genomes, 2 diploid cotton genomes (Gossypium raimondii and Gossypium arboreum) and 1 tetraploid cotton genome (Gossypium hirsutum) were downloaded for phylogenetic analysis and orthogroup identification.The promoter sequences of the CIPK6 genes (1000 bp upstream of the ATG initiation codon) were obtained from the cotton genome CottonFGD (https://cottonfgd.org/) and CottonMD (https://yanglab.hzau.edu.cn/CottonMD).
Data analysis	The species tree of 25 plants (except Gossypium hirsutum) was presented using the TimeTree Website (http://timetree.org/). The timing of WGDs were collected from the literature56, and marked on the species tree. OrthoFinder (v2.5.4) 57, with default parameters, was used to identify CIPK6 gene family members among 26 plants.The linkage plot between 2 diploid cotton and 1 tetraploid cotton and analysis of cis-acting elements were performed by TBtools (v1.123).

For manuscripts utilizing custom algorithms or software that are central to the research but not yet described in published literature, software must be made available to editors and reviewers. We strongly encourage code deposition in a community repository (e.g. GitHub). See the Nature Portfolio guidelines for submitting code & software for further information.

Data

Policy information about availability of data

All manuscripts must include a data availability statement. This statement should provide the following information, where applicable:

- Accession codes, unique identifiers, or web links for publicly available datasets
- A description of any restrictions on data availability
- For clinical datasets or third party data, please ensure that the statement adheres to our policy

Provide your data availability statement here.

Research involving human participants, their data, or biological material

Policy information about studies with human participants or human data. See also policy information about sex, gender (identity/presentation), and sexual orientation and race, ethnicity and racism.

Reporting on sex and gender	N/A
Reporting on race, ethnicity, or other socially relevant groupings	N/A
Population characteristics	N/A
Recruitment	N/A
Ethics oversight	N/A

Note that full information on the approval of the study protocol must also be provided in the manuscript.

Field-specific reporting

Please select the one below that is the best fit for your research. If you are not sure, read the appropriate sections before making your selection.

☒ Life sciences ☐ Behavioural & social sciences ☐ Ecological, evolutionary & environmental sciences

For a reference copy of the document with all sections, see nature.com/documents/nr-reporting-summary-flat.pdf

Life sciences study design

All studies must disclose on these points even when the disclosure is negative.

Sample size	1.The number of samples for species evolution is determined by the number of representative and reported species in evolutionary history. 2. The CIPK gene number is because there are only 8 in upland cotton. 3.The number of samples for data analysis is determined according to the specific number of samples collected. Sample size was described in each Figure legend.
Data exclusions	No data was excluded from the analysis.
Replication	All experiments were independently and successfully repeated for at least three times.
Randomization	These samples do not require randomization.
Blinding	These samples do not require blinding.

Reporting for specific materials, systems and methods

We require information from authors about some types of materials, experimental systems and methods used in many studies. Here, indicate whether each material, system or method listed is relevant to your study. If you are not sure if a list item applies to your research, read the appropriate section before selecting a response.

Materials & experimental systems

n/a	Involved in the study	
☐	☒	Antibodies
☐	☒	Eukaryotic cell lines
☒	☐	Palaeontology and archaeology
☒	☐	Animals and other organisms
☒	☐	Clinical data
☒	☐	Dual use research of concern
☐	☒	Plants

Methods

n/a	Involved in the study	
☒	☐	ChIP-seq
☒	☐	Flow cytometry
☒	☐	MRI-based neuroimaging

Antibodies

Antibodies used

The anti-His antibody was purchased form abclonal cat#AE003 and anti-GST antibody was purchased form abclonal cat#AE001. HRP Goat Anti-Mouse IgG (H+L) was purchased form abclonal cat#AS003.
All antibodies were used at 1:5000 dilution.

Validation

anti-His antibody: https://abclonal.com.cn/catalog/AE003
anti-GST antibody: https://abclonal.com.cn/catalog/AE001
HRP Goat Anti-Mouse IgG (H+L): https://abclonal.com.cn/catalog/AS003

Eukaryotic cell lines

Policy information about cell lines and Sex and Gender in Research

Cell line source(s)	Yeast strain Y2HGold (Clontech, Japan, 630498) and Y187 (Clontech, Japan, 630457).
Authentication	None of the cell lines used were authenticated.
Mycoplasma contamination	None of the cell lines used were authenticated.
Commonly misidentified lines (See ICLAC register)	No misidentified lines were used in this study.

Dual use research of concern

Policy information about dual use research of concern

Hazards

Could the accidental, deliberate or reckless misuse of agents or technologies generated in the work, or the application of information presented in the manuscript, pose a threat to:

No	Yes	
☒	☐	Public health
☒	☐	National security
☒	☐	Crops and/or livestock
☒	☐	Ecosystems
☒	☐	Any other significant area

Experiments of concern

Does the work involve any of these experiments of concern:

No	Yes	
☒	☐	Demonstrate how to render a vaccine ineffective
☒	☐	Confer resistance to therapeutically useful antibiotics or antiviral agents
☒	☐	Enhance the virulence of a pathogen or render a nonpathogen virulent
☒	☐	Increase transmissibility of a pathogen
☒	☐	Alter the host range of a pathogen
☒	☐	Enable evasion of diagnostic/detection modalities
☒	☐	Enable the weaponization of a biological agent or toxin
☒	☐	Any other potentially harmful combination of experiments and agents

Plants

Seed stocks	Seeds of cotton (Gossypium hirsutum cv. Jin668 and YZ1) from our lab.
Novel plant genotypes	Overexpression plants were generated by 35s promoter-driven pK2GW7 and obtained by Agrobacterium transformation. Mutant plants were obtained by CRISPR-Cas 9-mediated gene editing.
Authentication	The overexpression plants were authenticated by molecular testing. Mutant plants were authenticated by the editing efficiency assay (Hi-Tom).
